# Risk factors, survival analysis, and nomograms for high-grade endometrial stromal sarcoma patients with distant metastasis: a population-based study (2010–2019)

**DOI:** 10.3389/fonc.2025.1567195

**Published:** 2025-03-07

**Authors:** Cheng Wang, Dongni Liang, Wei Kuang, Huanxin Sun, Yuling Kou, Wei Wang, Jing Zeng

**Affiliations:** ^1^ Department of Pathology, West China Second University Hospital of Sichuan University, Chengdu, Sichuan, China; ^2^ Key Laboratory of Birth Defects and Related Diseases of Women and Children (Sichuan University), Ministry of Education, Chengdu, Sichuan, China; ^3^ Department of Gynecology and Obstetrics, West China Second University Hospital, Sichuan University, Chengdu, Sichuan, China

**Keywords:** high-grade endometrial stromal sarcoma (HGESS), distant metastasis, nomogram, overall survival, prognosis factors

## Abstract

**Background:**

High-grade endometrial stromal sarcoma (HGESS) is a rare, aggressive malignant tumor that often metastasizes early and is associated with a poor prognosis. This study aimed to develop a nomogram to predict the risk factors for distant metastases and the prognostic factors at the time of initial diagnosis.

**Methods:**

Data on patients diagnosed with HGESS from 2010 to 2019 were extracted from the Surveillance, Epidemiology, and End Results (SEER) database. Patients were randomly divided into the training and validation sets. Univariate and multivariate regression analyses were conducted to identify significant independent risk factors for distant metastases in HGESS patients, and univariate and multivariate Cox regression analyses were used to identify prognostic factors of HGESS patients with distant metastases. The Akaike information criterion (AIC) was used to further refine variables and construct a nomogram for predicting overall survival (OS) of HGESS patients with distant metastases. Two nomograms were developed and evaluated using receiver operating characteristic (ROC) curves, calibration plots, decision curves analysis, and concordance-index (C-index). In addition, Kaplan-Meier (KM) analysis was performed to evaluate OS in both the entire cohort and the metastasis cohort.

**Results:**

A total of 360 HGESS patients were included, of whom 89 patients (24.7%) had distant metastases at initial diagnosis. Risk factors for distant metastases in HGESS patients included race, tumor size, T stage, and N stage. Prognostic factors for distant metastasis in HGESS patients included N stage and systemic therapy. Three variables - age, N stage and systemic therapy - were incorporated to construct the nomogram for predicting prognosis. The C-indexes for the training and validation sets were 0.776 and 0.710, respectively. In the entire cohort, significant differences in median OS were observed for tumor size, Federation International of Gynecology and Obstetrics (FIGO) stage, number of nodes examined, surgery, and radiotherapy. In metastasis cohort, significant differences in median OS were observed for N stage, surgery, chemotherapy, and systemic therapy.

**Conclusions:**

The two nomograms developed in this study accurately predict the occurrence and prognosis of HGESS patients with distant metastases, which may aid clinical decision-making.

## 1 Introduction

Endometrial stromal sarcoma (ESS), representing < 1% of uterine malignancies, is classified by the 2020 World Health Organization (WHO) into low-grade endometrial stromal sarcoma (LGESS) and high-grade endometrial stromal sarcoma (HGESS) subtypes (1, 2). LGESS typically presents with tongue-shaped and island-shaped infiltration, usually accompanied by lymphovascular invasion (LVSI). Common molecular alterations include JAZF1::SUZ12, JAZF1::PHF1, MEAF6::PHF1, and EPC1::PHF1 gene fusions (3). It generally has a relatively good prognosis, with late recurrence. In contrast, HGESS exhibits aggressive features including nuclear atypia, uniform high-grade round or spindle cell histology, a fine vascular network, and tongue-shaped infiltration into the myometrium. HGESS is associated with characteristic molecular alterations such as YWHAE::NUTM2 gene fusion, BCOR rearrangement, or internal tandem duplication of BCOR (4–7). There are few reports in the literature that describe the treatment and prognosis of HGESS, and even fewer regarding recurrence or metastasis. As a result, reaching a consensus on the optimal treatment for this disease has been challenging.

Previous studies have identified a series of factors (such as age, Federation International of Gynecology and Obstetrics (FIGO) stage, grade, tumor size, surgery, and treatment methods) that are correlated with predicting the survival risk of uterine sarcoma (8, 9). However, there is limited reliable information on individual prediction models to evaluate the prognosis of HGESS with metastasis. Using SEER data (2000–2019), we developed dual nomograms to predict metastasis risk and overall survival (OS) in metastatic HGESS, addressing critical gaps in clinical decision-making.

## 2 Materials and methods

### 2.1 Patient selection

**Figure 1:**
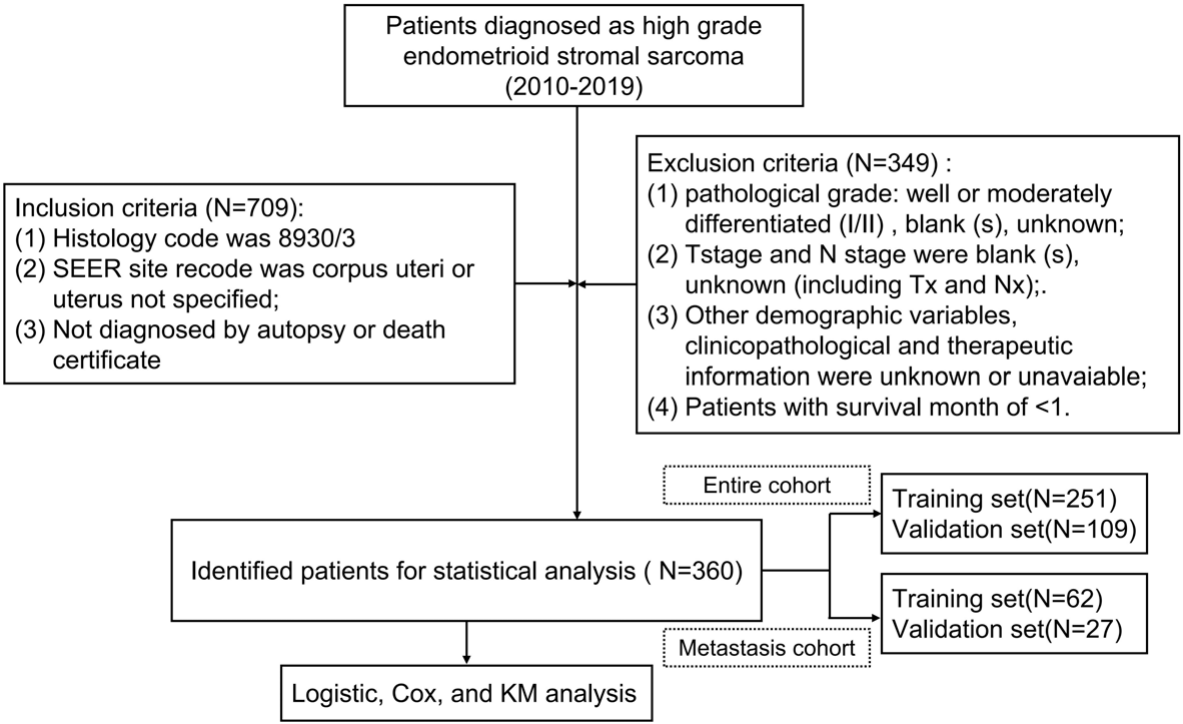
The study flow diagram of the selection process and detailed workflow.

SEER*Stat software (version 8.3.5) was used to download data for female patients diagnosed with HGESS from the SEER 18 database between 2000 and 2019, the largest National Cancer Database in the United States. The inclusion criteria were as follows: (i) pathological code 8930/3; (ii) SEER site recodes including corpus uteri or uterus not specified; (iii) histological confirmation of HGESS as the primary tumor. The exclusion criteria were: (i) pathological grade classified as well or moderately differentiated (I/II), blank (s), and unknown; (ii) missing information on AJCC stage; (iii) patients without follow-up data or with survival time of less than one month; (iv) missing demographic variables and clinicopathological information. According to the 2009 FIGO stage criteria, T1-T4 stage corresponding to FIGO stage I-IV, N1 stage corresponding to FIGO stage III, and M1 corresponding to FIGO stage IV. The selection process and detailed workflow of this study are illustrated in **Figure 1**. A total of 360 patients diagnosed with HGESS were included in this study, including 89 patients with distant metastases. The entire cohort was used as the diagnostic cohort to identify the risk factors for distant metastases and to establish a predictive nomogram. The prognostic cohort was used to study prognostic factors for patients with distant metastasis and develop a prognostic nomogram.

### 2.2 Study variables and data collection

In the diagnostic cohort, all patients were randomly assigned to the training and validation sets in a 7:3 ratio using R software. In the prognostic cohort, patients in the training and validation sets were selected from the distant metastasis group in the corresponding sets of the diagnostic cohort. The training set was used to screen variables and construct the nomogram, while the validation set was used to validate it using corresponding patients. The following variables were included for identifying the risk factor for distant metastases in HGESS patients: age at diagnosis, race, marital status, tumor size, T stage, and N stage.

Additionally, we incorporated additional variables to investigate prognostic factors for HGESS patients with distant metastases: surgery type, radiotherapy, chemotherapy, and systemic therapy. The surgery types contained hysterectomy with bilateral salpingectomy and ovariectomy oophorectomy (Hys + BSO); hysterectomy without bilateral salpingectomy and ovariectomy oophorectomy (Hys – BSO); local surgery, exenteration, and other unspecified surgery type(L/E/O); and no surgery. In this study, OS was the primary endpoint of interest, defined as the time interval from the date of diagnosis to the date of death from any cause.

### 2.3 Statistical analysis

Chi-square test or Fisher’s exact test were used to compare the distribution of variables between the two sets. The data were presented as frequencies and percentages. Univariate logistic analysis was performed for categorical variables to identify risk factors for distant metastasis. Variables with a P<0.05 in the univariate analysis were further selected for the multivariate logistic regression to identify independent risk factors for distant metastasis in HGESS patients. Based on these independent risk factors, a diagnostic nomogram was developed. The receiver operating characteristic (ROC) curve of the nomogram was plotted, and the corresponding area under the curve (AUC) was calculated to assess the nomogram’s discrimination ability. Calibration curves and decision curve analysis (DCA) were also performed to evaluate the performance of the nomogram.

For prognostic factors, univariate and multivariate Cox regression analyses were conducted to determine independent prognostic factors for HGESS patients with distant metastases. Stepwise regression was used to identify variables for inclusion in the prognostic nomogram (10). A prognostic nomogram based on the independent prognostic factors was developed to predict 1-year and 3-year OS of HGESS patients with distant metastases. The concordance index (C-index) and calibration curve were also used to evaluate discriminative ability. Kaplan-Meier plots of OS were generated. In this study, a P value <0.05 was considered statistically significant. C-index and AUC values greater than 0.7 were considered indictive of good predictive performance. Additionally, the variance inflation factor (VIF) was assessed for the covariates in the nomogram, and a VIF > 4.0 indicated multicollinearity. Variables with VIF < 4.0 were included in the final model analysis.

## 3 Results

### 3.1 Baseline characteristics of the entire cohort

A total of 360 patients diagnosed with HGESS were enrolled for statistical analysis based on the inclusion and exclusion criteria and were randomly divided into a training set of 251 patients and a validation set of 109 patients at a 7:3 ratio. The median age of the entire cohort was 59 years old (ranging from 19 to 95), with the majority being of white ethnicity (76.9%). The majority were diagnosed at T1-T2 (76.1%) and N0 (82.8%) stage. Approximately one-third of the patients had distant metastasis (corresponding to FIGO stage IV) at the time of diagnosis. Most patients (72.2%) had a tumor size greater than 5cm at diagnosis, except for cases with unspecified data. Regarding treatment, more than two-thirds (72.8%) of patients underwent Hys + BSO. Half of the patients received lymph node examination and chemotherapy, but only 22.2% received radiotherapy. In addition, more than half of patients received comprehensive systemic therapy. Overall, patients with HGESS had a high (67.2%) mortality rate, with median survival time of 12.5 months (range: 10 to 15, 95% CI). The training and validation sets were comparable in terms of demographic and clinical features. The demographic and clinicopathological information of all HGESS patients is shown in **Table 1**.

**Table 1:** Demographic and clinical characteristics of patients diagnosed with high-grade endometrial stromal sarcoma.

	Overall	Training	Validation	
	(N=360)	(N=251)	(N=109)	*p*
Age				0.167
<45	39 (10.8%)	30 (12.0%)	9 (8.3%)	
45-55	103 (28.6%)	77 (30.7%)	26 (23.9%)	
>55	218 (60.6%)	144 (57.4%)	74 (67.9%)	
Race				0.641
Asian/Pacific Islander/Other	26 (7.2%)	20 (8.0%)	6 (5.5%)	
Black	57 (15.8%)	38 (15.1%)	19 (17.4%)	
White	277 (76.9%)	193 (76.9%)	84 (77.1%)	
Marital status				0.251
Married	180 (50.0%)	131 (52.2%)	49 (45.0%)	
Single/Widowed/Other	180 (50.0%)	120 (47.8%)	60 (55.0%)	
Tumor size				0.319
<5cm	52 (14.4%)	39 (15.5%)	13 (11.9%)	
5-10cm	124 (34.4%)	80 (31.9%)	44 (40.4%)	
>10cm	136 (37.8%)	95 (37.8%)	41 (37.6%)	
Not specified	48 (13.3%)	37 (14.7%)	11 (10.1%)	
T				0.142
T1-T2	274 (76.1%)	197 (78.5%)	77 (70.6%)	
T3-T4	86 (23.9%)	54 (21.5%)	32 (29.4%)	
N				0.257
N0	298 (82.8%)	212 (84.5%)	86 (78.9%)	
N1	62 (17.2%)	39 (15.5%)	23 (21.1%)	
Metastasis status				0.807
Multiple (Liver/Brain/Bone/Lung)	11 (3.1%)	9 (3.6%)	2 (1.8%)	
Single (Liver/Brain/Bone/Lung)	42 (11.7%)	31 (12.4%)	11 (10.1%)	
Non-Liver/Brain/Bone/Lung	36 (10.0%)	25 (10.0%)	11 (10.1%)	
None	271 (75.3%)	186 (74.1%)	85 (78.0%)	
FIGO				0.195
I	149 (41.4%)	111 (44.2%)	38 (34.9%)	
II	40 (11.1%)	28 (11.2%)	12 (11.0%)	
III	73 (20.3%)	44 (17.5%)	29 (26.6%)	
IV	98 (27.2%)	68 (27.1%)	30 (27.5%)	
Number of nodes examined				0.195
>12	81 (22.5%)	50 (19.9%)	31 (28.4%)	
≤12	103 (28.6%)	73 (29.1%)	30 (27.5%)	
None	176 (48.9%)	128 (51.0%)	48 (44.0%)	
Surgery				0.933
Hys-BSO	16 (4.4%)	12 (4.8%)	4 (3.7%)	
Hys+BSO	262 (72.8%)	183 (72.9%)	79 (72.5%)	
Not specified	59 (16.4%)	41 (16.3%)	18 (16.5%)	
No surgery	23 (6.4%)	15 (6.0%)	8 (7.3%)	
Chemotherapy				0.427
No	155 (43.1%)	112 (44.6%)	43 (39.4%)	
Yes	205 (56.9%)	139 (55.4%)	66 (60.6%)	
Radiotherapy				0.530
No	280 (77.8%)	198 (78.9%)	82 (75.2%)	
Yes	80 (22.2%)	53 (21.1%)	27 (24.8%)	
Systemic therapy				0.904
No	152 (42.2%)	107 (42.6%)	45 (41.3%)	
Yes	208 (57.8%)	144 (57.4%)	64 (58.7%)	
Number of events	242 (67.2%)	168 (66.9%)	74 (67.9%)	0.956

Hys-BSO, hysterectomy and without bilateral salpingectomy and ovariectomy oophorectomy; Hys+BSO, hysterectomy with bilateral salpingectomy and ovariectomy.

### 3.2 Incidence and risk factors of distant metastases in HGESS patients, and construction and validation of the diagnostic nomogram

**Figure 2:**
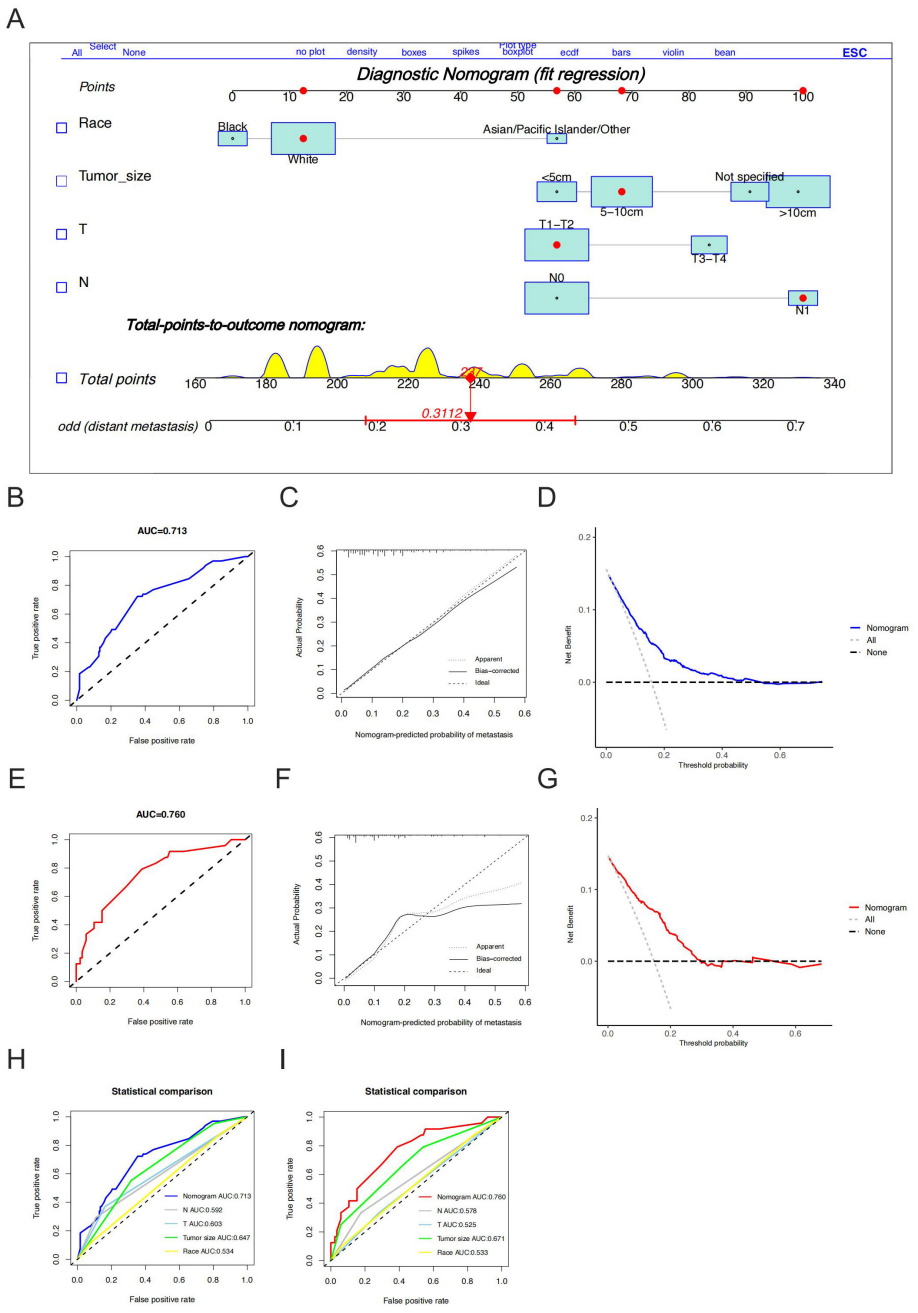
Construction and validation of a diagnostic nomogram. A nomogram for predicting the risk of distant metastases in high-grade endometrial stromal sarcoma **(A)**. The receiver operating characteristic curve **(B)**, calibration curve **(C)** and decision curve analysis **(D)** of the training set, and the receiver operating characteristic curve **(E)**, calibration curve **(F)**, and decision curve analysis **(G)** of the validation set, comparison of area under the receiver operating characteristic curves between nomogram and all independent factors (including race, tumor size, T stage and N stage) in the training set **(H)** and Validation set **(I)**.

Eighty-nine patients (24.7%) had metastases at the initial diagnosis, while 271 patients (75.3%) did not. Based on the occurrence of metastasis in HGESS patients, univariate logistic regression analysis identified race, tumor size, T stage, and N stage were the potential risk factors (**Table 2**). Furthermore, multivariate logistic regression analysis revealed four independent risk predictors for distant metastasis in primary HGESS patients: race, tumor size, T stage, and N stage. We constructed a nomogram to predict the risk of distant metastases in HGESS patients these four independent predictors (**Figure 2A**). ROC curves were generated for both the training and validation sets, with AUC values of 0.713 and 0.760, respectively (**Figures 2B, E**). Meanwhile, ROC curves for all independent predictors were also generated (**Figures 2H, I**), showing better discriminative ability than any individual factors in both the training and validation sets. The calibration curves of the nomogram demonstrated excellent consistency between the observed and predicted results (**Figures 2C, F**). Additionally, DCA curves (**Figures 2D, G**) indicated that the diagnostic nomogram is a precise tool for assessing distant metastasis risk.

**Table 2:** Univariate and multivariate logistic analyses of distant metastasis in high-grade endometrial stromal sarcoma patients.

	Univariate analysis		Multivariate analysis	
	OR (95%CI)	*p*	OR (95%CI)	*p*
Age				
<45	1			
45-55	1.014 (0.524-2.014)	0.973		
>55	0.585 (0.313-1.124)	0.164		
Race				
Asian/Pacific Islander/Other	1		1	
Black	0.326 (0.137-0.765)	0.031	0.786 (0.668-0.925)	0.015
White	0.435 (0.219-0.882)	0.048	0.828 (0.719-0.953)	0.028
Marital status				
Married	1			
Single/Widowed/Other	0.811 (0.541-1.213)	0.393		
Tumor size				
<5cm	1		1	
5-10cm	1.746 (0.809-4.153)	0.257	1.049 (0.9364-1.176)	0.486
>10cm	3.918 (1.895-9.063)	0.003	1.197 (1.0664-1.3425)	0.011
Not specified	3.157 (1.340-8.006)	0.032	1.154 (1.005-1.326)	0.089
T				
T1-T2	1		1	
T3-T4	2.256 (1.447-3.502)	0.002	1.120 (1.027-1.221)	0.032
N				
N0	1		1	
N1	1.255 (1.139-1.384)	<0.001	1.200 (1.090-1.323)	0.002

### 3.3 Baseline characteristics of the metastasis cohort

The study used data from 89 eligible HGESS patients with distant metastases to explore prognostic factors (**Table 3**). The median age of these patients was 56 years, with the majority being white (75.3%), diagnosed at T1-T2 (64%) and N0 (69.7%) stage. Excluding cases with unspecified data, most (61.3%) patients had a tumor size greater than 10 cm and had metastases at the time of the initial diagnosis. Among these patients, 59.6% had metastases in one of the following organs: liver, brain, bone or lung. Forty-seven percent had single metastases, while 12.4% had multiple metastases. Regarding treatment, the majority of patients underwent surgery (85.4%), chemotherapy (66.3%) and systemic therapy (62.9%), but only a minority received radiotherapy (15.7%) or lymph nodes examination (27%). The Chi-square test and Fisher’s exact test indicated no significant differences in variables between the training and validation set. Overall, HGESS patients with distant metastases had a high mortality rate (88.8%), with a median survival time of 6 months (range: 3 to 7, 95% CI).

**Table 3:** Baseline characteristics of high-grade endometrial stromal sarcoma patients with distant metastasis.

	Overall	Training	Validation	
	(N=89)	(N=62)	(N=27)	p
Age				0.424
<45	12 (13.5%)	7 (11.3%)	5 (18.5%)	
45-55	32 (36.0%)	21 (33.9%)	11 (40.7%)	
>55	45 (50.6%)	34 (54.8%)	11 (40.7%)	
Race				0.370
Asian/Pacific Islander/Other	11 (12.4%)	6 (9.7%)	5 (18.5%)	
Black	11 (12.4%)	7 (11.3%)	4 (14.8%)	
White	67 (75.3%)	49 (79.0%)	18 (66.7%)	
Marital status				0.174
Married	48 (53.9%)	30 (48.4%)	18 (66.7%)	
Single/Widowed/Other	41 (46.1%)	32 (51.6%)	9 (33.3%)	
Tumor size				0.207
<5cm	6 (6.7%)	4 (6.5%)	2 (7.4%)	
5-10cm	23 (25.8%)	15 (24.2%)	8 (29.6%)	
>10cm	46 (51.7%)	36 (58.1%)	10 (37.0%)	
Not specified	14 (15.7%)	7 (11.3%)	7 (25.9%)	
T				0.920
T1-T2	57 (64.0%)	39 (62.9%)	18 (66.7%)	
T3-T4	32 (36.0%)	23 (37.1%)	9 (33.3%)	
N				1
N0	62 (69.7%)	43 (69.4%)	19 (70.4%)	
N1	27 (30.3%)	19 (30.6%)	8 (29.6%)	
Metastasis status				0.056
Multiple (Liver/Brain/Bone/Lung)	11 (12.4%)	4 (6.5%)	7 (25.9%)	
Single (Liver/Brain/Bone/Lung)	42 (47.2%)	31 (50.0%)	11 (40.7%)	
Non-Liver/Brain/Bone/Lung	36 (40.4%)	27 (43.5%)	9 (33.3%)	
Number of nodes examined				0.319
>12	10 (11.2%)	6 (9.7%)	4 (14.8%)	
≤12	14 (15.7%)	12 (19.4%)	2 (7.4%)	
None	65 (73.0%)	44 (71.0%)	21 (77.8%)	
Surgery				0.202
No	13 (14.6%)	7 (11.3%)	6 (22.2%)	
Yes	76 (85.4%)	55 (88.7%)	21 (77.8%)	
Chemotherapy				0.495
No	30 (33.7%)	19 (30.6%)	11 (40.7%)	
Yes	59 (66.3%)	43 (69.4%)	16 (59.3%)	
Radiotherapy				0.212
No	75 (84.3%)	50 (80.6%)	25 (92.6%)	
Yes	14 (15.7%)	12 (19.4%)	2 (7.4%)	
Systemic therapy				0.096
No	33 (37.1%)	19 (30.6%)	14 (51.9%)	
Yes	56 (62.9%)	43 (69.4%)	13 (48.1%)	
Number of events	79 (88.8%)	54 (87.1%)	25 (92.6%)	0.717

Hys-BSO, hysterectomy and without bilateral salpingectomy and ovariectomy oophorectomy; Hys+BSO, hysterectomy with bilateral salpingectomy and ovariectomy.

### 3.4 Construction of the prognostic nomogram for predicting OS in metastatic patients

**Figure 3:**
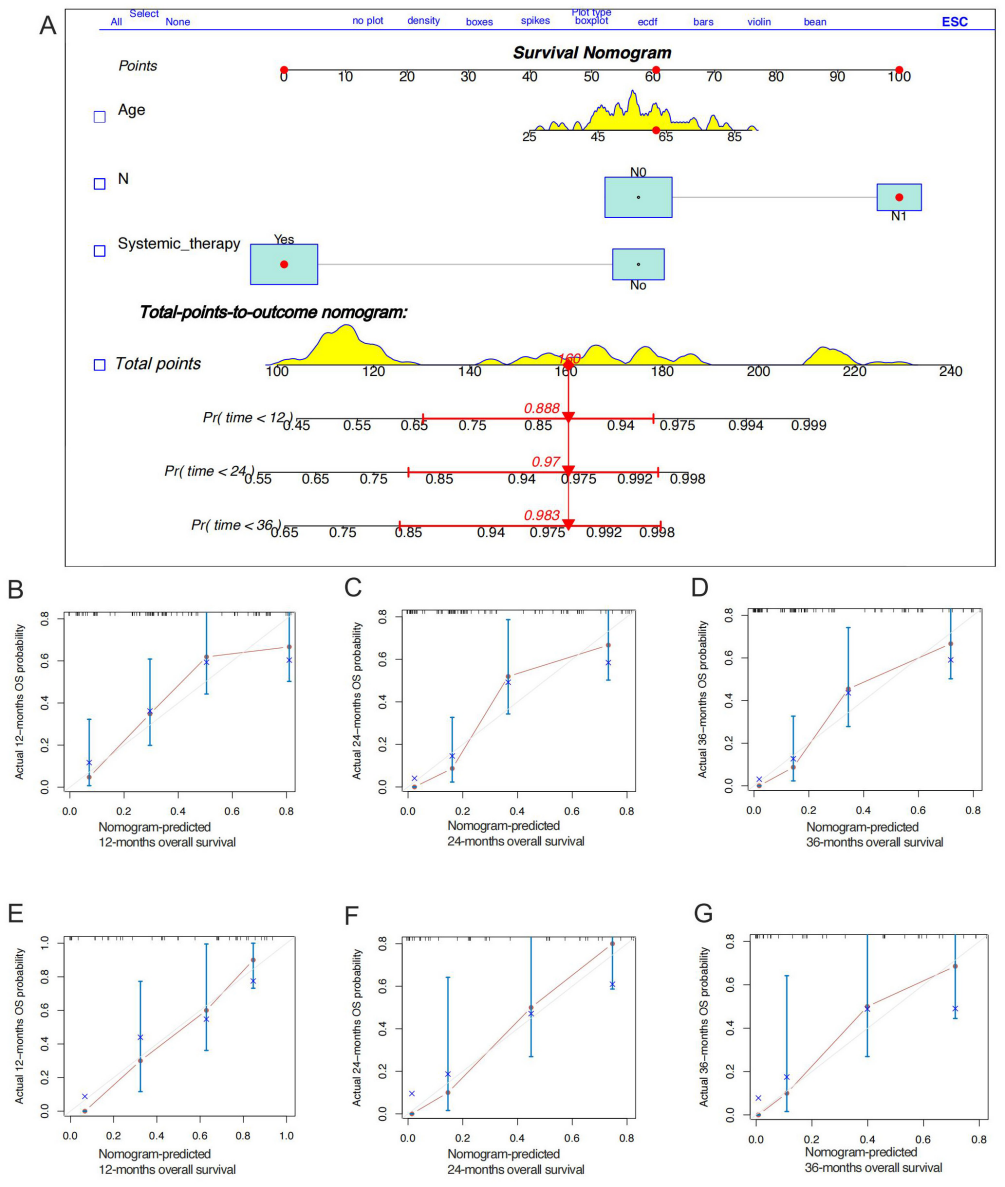
Construction of a prognostic nomogram for predicting the overall survival of high-grade endometrial stromal sarcoma patients with distant metastases for the 12, 24, and 36 months **(A)**. The calibration curves of the prognostic nomogram for the 12, 24, and months in the training set **(B-D)** and the validation set **(E-G)**, respectively.

Stepwise regression results indicated no collinearity among the screened variables (VIF<4), including N stage and systemic therapy. Univariate and multivariate Cox regression analyses were performed to identify robust prognostic factors, revealing that the N stage (p<0.001) and systemic therapy (p=0.004) were independent prognostic factors for HGESS patients with distant metastases (**Table 4**). Based on these prognostic factors, a nomogram was developed to predict the OS of HGESS patients with distant metastases (**Figure 3A**). The nomogram showed that prognosis was most influenced by N status, and the total score was calculated using individual factors. Most patients in this study had total risk points ranging from 100 to 220. The C-index was 0.776 for the training set and 0.710 in the validation set. Calibration curves for predicting 12 months, 24months and 36 months OS exhibited a strong agreement between nomogram-predicted OS and actual outcomes in both the training set (**Figures 3B–D**) and validation set (**Figures 3E–G**).

**Table 4:** Univariate and multivariate Cox analyses of distant metastasis in high-grade endometrial stromal sarcoma patients.

	Univariate analysis		Multivariate analysis	
	OR (95%CI)	*p*	OR (95%CI)	*p*
Age				
<45	1			
45-55	1.088 (0.528-2.241)	0.819		
>55	1.569 (0.785-3.138)	0.202		
Race				
Asian/Pacific Islander/Other	1		1	
Black	2.508 (1.024-6.143)	0.0443	2.029 (0.764-5.378)	0.156
White	1.214 (0.601-2.453)	0.5881	1.344 (0.646-2.799)	0.429
Marital status				
Married	1			
Single/Widowed/Other	1.073 (0.689-1.672)	0.755		
Tumor size				
<5cm	1		1	
5-10cm	2.118 (0.724-6.195)	0.1704	1.922 (0.652-5.673)	0.237
>10cm	1.632 (0.583-4.566)	0.3511	1.836 (0.630-5.349)	0.265
Not specified	3.621 (1.1821-11.094)	0.0243	2.413 (0.742-7.849)	0.143
T				
T1-T2	1			
T3-T4	0.996 (0.627-1.581)	0.986		
N				
N0	1		1	
N1	2.594 (1.585-4.244)	<0.001	3.100 (1.761-5.459)	<0.001
Metastasis status				
Multiple (Liver/Brain/Bone/Lung)	1			
Single (Liver/Brain/Bone/Lung)	0.653 (0.323-1.319)	0.235		
Non-Liver/Brain/Bone/Lung	0.898 (0.439-1.833)	0.767		
Number of nodes examined				
>12	1			
≤12	1.417 (0.576-3.488)	0.448		
None	1.617 (0.769-3.398)	0.205		
Surgery				
No	1		1	
Yes	0.483 (0.264-0.882)	0.0178	1.77635 (0.696-4.532)	0.229
Chemotherapy				
No	1		1	
Yes	0.356 (0.221-0.576)	<0.001	0.954 (0.424-2.147)	0.909
Radiotherapy				
No	1			
Yes	0.707 (0.374-1.338)	0.287		
Systemic therapy				
No	1		1	
Yes	0.255 (0.155-0.418)	<0.001	0.254 (0.100-0.641)	0.004

Hys-BSO, hysterectomy and without bilateral salpingectomy and ovariectomy oophorectomy; Hys+BSO, hysterectomy with bilateral salpingectomy and ovariectomy.

### 3.5 OS depicted by Kaplan-Meier plots

**Figure 4:**
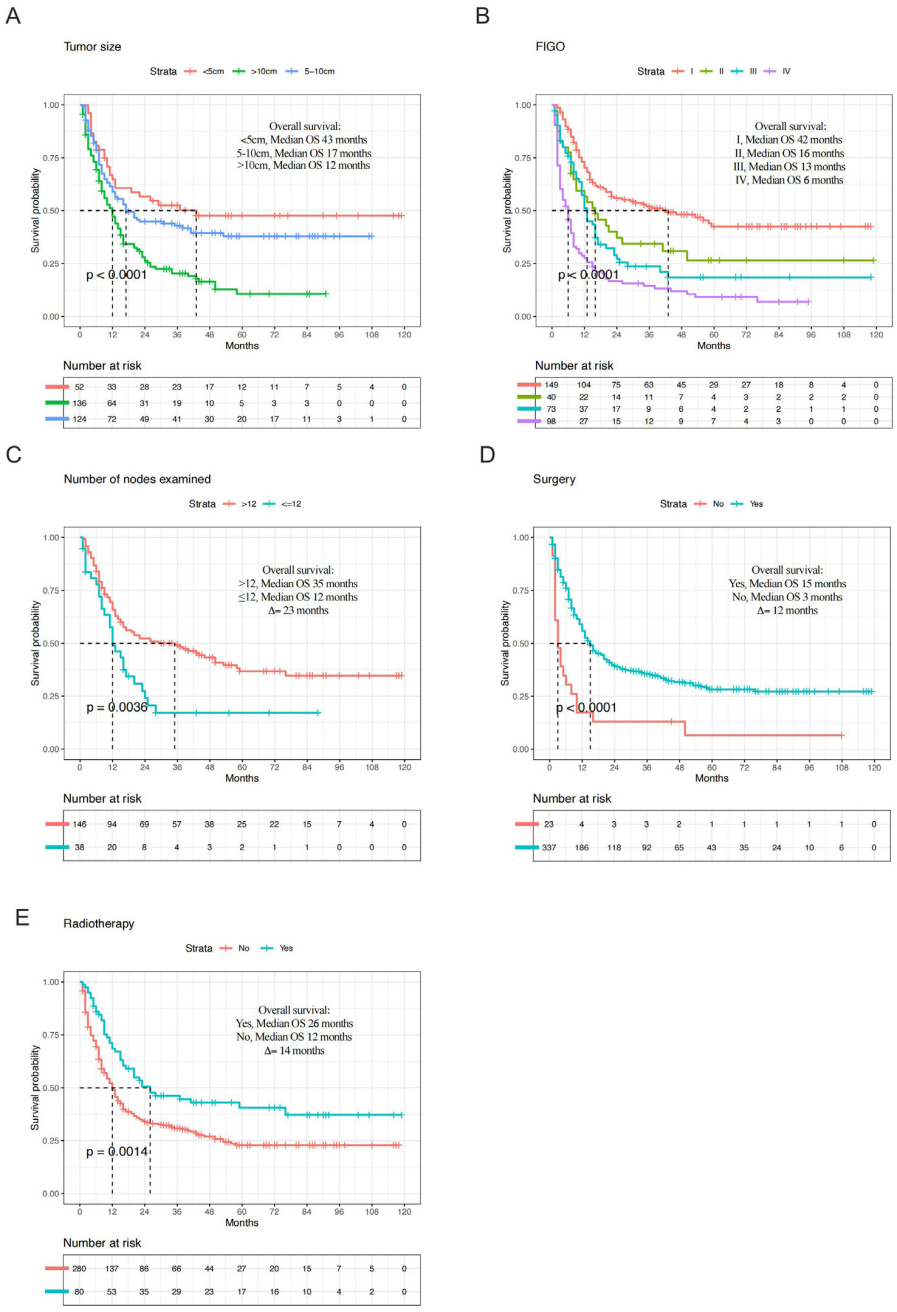
Kaplan-Meier plots depicting overall survival in high-grade endometrial stromal sarcoma (entire cohort) of tumor size **(A)**, Federation International of Gynecology and Obstetrics (FIGO) stage **(B)**, number of nodes examined **(C)**, surgery **(D)**, and radiotherapy **(E)**.

In the entire cohort, the median OS in tumor size > 10 cm group was 12 months, compared to 43 months in the tumor size ≤ 5 cm group (Δ = 31 months; p < 0.001, **Figure 4A**). The median OS for different FIGO stage was 42 months for stage I, 16 months for stage II, 13 months for stage III, and 6 months for stage IV (p < 0.001, **Figure 4B**). In terms of clinical treatment, the median OS in the group with more than 12 nodes examined was 35 months, compared to 12 months in the group with fewer than 12 nodes examined (Δ = 23 months; p=0.004, **Figure 4C**). Additionally, OS was statistically significant for surgery (p < 0.001, **Figure 4D**) and radiotherapy (p =0.001, **Figure 4E**). In metastasis cohort, median OS in N0 group was 7 months, compared to 3 months in the N1 group (Δ = 4 months; p< 0.001, **Supplementary Figure S1A**). OS was also statistically significant for surgery (Δ = 4 months; p=0.02, **Figure 4D**), chemotherapy (Δ = 4 months; p< 0.001, **Figure 4D**), and systemic therapy (Δ = 6 months; p< 0.001, **Figure 4D**).

## 4 Discussion

In the current study, we constructed a diagnostic nomogram for predicting distant metastases in patients with diagnosed HGESS and a prognostic nomogram for patients with distant metastases. By obtaining data on several vital variables in the nomograms, diagnosis-related and prognosis-related scores can be calculated, which can guide further clinical assessment and intervention. This model demonstrated a higher predictive capacity for the risk of distant metastases and performed excellently through calibration curves, ROC curves, and DCA. It may improve the status of risk assessment and enable more accurate personalized clinical decision-making.

HGESS recurs and metastasizes much earlier, usually within one year, with median progression-free survival and OS of 7-11 months and 11-23 months, respectively (11, 12). Due to the poor prognosis of HGESS patients with distant metastases, the early detection of distant metastases is crucial to ensure that patients receive appropriate systemic treatment. Numerous independent risk factors affect OS in HGESS, including age, race, marital status, tumor size, disease stage, lymphadenectomy, radiotherapy and chemoradiotherapy (13). An analysis of a population-based study of pan-soft tissue sarcomas showed that patients with larger tumors located in the head and neck, retroperitoneum, and certain specific pathological subtypes, such as ESS, had a high risk of lymph node metastasis (14). In this study, we used the most recent large sample with comprehensive clinical information from the SEER database (2010–2019), which showed a higher metastasis rate of 24.7% for HGESS, similar to that previous studies (3, 15, 16). We screened four prognostic factors (namely, race, tumor size, T stage, and N stage), which were incorporated into a diagnosis nomogram to predict the risk of distant metastases in HGESS.

Most patients with soft tissue sarcoma die from their disease within 12-15 months of diagnosis of advanced disease, with a 5-year OS rate approaching 8% (17). Currently, surgical resection is the standard for HGESS, supplemented by systemic therapy and local radiotherapy (18). The standard procedure is total hysterectomy ± bilateral salpingectomy and ovariectomy oophorectomy (19). However, the efficacy of lymph node dissection for HGESS is controversial. According to our data, the lymph node positive rate of HGESS was 17.2%, slightly lower to that previously reported, which may be related to the year of diagnosis, as we included data from the last 10 years of HGESS diagnosis (5, 7). Systematic lymph node dissection does not provide a survival benefit in those with significantly enlarged or metastatic lymph nodes. However, it has also been documented that early complete surgical resection, including lymph node dissection, can benefit patients (5). In term of the number of lymph nodes examined, we tested for OS difference between more than 12 nodes examined and less than 12 nodes examined in entire cohort (35 months vs 12 months, p<0.01), but not statistically difference in the metastatic cohort (p>0.05), similar to that previously reported (11, 20). In patients undergoing initial surgery, lymph node dissection is usually not performed, but the presence of enlarged lymph nodes should be carefully examined intraoperatively. Lymphadenectomy is not recommended for patients with uterine sarcoma because lymph node metastasis is uncommon (18).

Surgery, chemotherapy and radiotherapy were not incorporated in the nomogram in our study because it increased the AIC value of the prognostic nomogram of metastasis cohort. This does not mean that these factors have no benefit for OS. Isolated or resected oligometastatic recurrent HGESS lesions should strive for surgical opportunities as much as possible, with emphasis on multidisciplinary combined resection, supplemented with postoperative radiotherapy and chemotherapy combination. For inoperable patients with extensive metastases, systemic therapy is the mainstay of treatment, supplemented with palliative radiotherapy if necessary (21). Radiotherapy has limited therapeutic effect on HGESS, and patients with high tumor mutation burden (TMB) who are surgically unresectable or have distant metastases may choose for immunotherapy in the absence of a satisfactory treatment (22). Recently, a single-central phase 2 showed that immunotherapy is increasingly used in patients with sarcomas, including those with solid tumors (23). Notably, we constructed a prognostic nomogram to predict the prognosis of HGESS patients with distant metastases and incorporated new factors that could be included in a prognostic nomogram, such as demographic and clinicopathological characteristics, into a quantitative model. Here, lymph status (N stage) was identified as an important contributor to the survival of HGESS metastases cohort. Although, the necessity and effectiveness of lymph node assessment in improving survival remains controversial (20, 24), this may provide some reference for surgeons, suggesting that lymph node dissection in patients with advanced HGESS may improve patient OS. Indeed, assessing the long-term efficacy and prognosis of HGESS patients requires individualized comprehensive analysis, including demographic and clinicopathological information.

For HGESS patients with distant metastases, mostly due to advanced disease, preoperative multidisciplinary collaboration is usually required for precise assessment and joint decision-making regarding the treatment plan, allowing systemic treatment to prolong OS in patients with distant metastases (median OS 8 months, p<0.01). For recurrent or metastatic cases, chemotherapy regimens such as doxorubicin, ifosfamide, gemcitabine, and docetaxel are commonly used. These treatments are often combined with surgery or radiation for debulking or palliative purposes, depending on the disease’s resectability (3). As an emerging and promising treatment, immune checkpoint inhibitors or targeted drugs have been shown to significantly prolong OS in patients with other gynecological cancers. Kang, et al. found that the majority (85.7%) of HGESS patients had positive predictors of immunotherapy efficacy, as well as high immune infiltration, especially in patients with ZC3H7B::BCOR fusion genome (22). Potential therapeutic targets for HGESS include platelet-derived growth factor receptor (PDGFR), human epidermal growth factor receptor-2 (HER-2), and c-Kit, most of which are in clinical trials (25). Patients with advanced relapse and failure of conventional therapy are recommended to undergo genetic testing and try individualized targeted therapy. Thus, future iterations of systemic therapy or individualized precision therapy may in fact demonstrate even greater potency.

Although the nomogram performed well, several potential imitations of this study need to be noted. Firstly, this is a retrospective study based on the SEER database. HGESS is a rare disease and the diagnosis is currently based on morphological and molecular features, but the SEER database lacks genomic and transcriptomic characterization data. Secondly, data on some clinicopathological parameters, such as surgical margin status, vascular invasion, and the detailed systemic treatments (hormone therapy, chemotherapy dose or regimens, and immunotherapy or targeted therapy), were unavailable in the SEER database. Thirdly, we lack sufficient data on Chinese patients to compare with those in the SEER database. Further multicenter clinical validation is required in the future, with comprehensive assessment of demographic characteristics as well as clinicopathological features, particularly surgical margin status, vascular invasion, hormonal therapy, immunotherapy and targeted therapy.

## 5 Conclusions

In summary, our study determined that race, tumor size, T and N stage were independent risk factors for HGESS patients with distant metastases, and age, N stage and systemic therapy were independent prognostic factors for distant metastases patients. The two nomograms can be used as individual and convenient tools for assessing the risk and prognosis of HGESS patients with distant metastases, which could help clinicians make better clinical decisions.

## Data Availability

Publicly available datasets were analyzed in this study. The dataset used to perform the statistical analysis is publicly available in the Surveillance Epidemiology, and End Results (SEER) database (http://seer.cancer.gov/).

## References

[1] Conklin CMJ Longacre TA. Endometrial stromal tumors: the new WHO classification. Adv Anat Pathol. (2014) 21:383–93. doi: 10.1097/PAP.0000000000000046 25299308

[2] Major FJ Blessing JA Silverberg SG Morrow CP Creasman WT Currie JL. Prognostic factors in early-stage uterine sarcoma. A Gynecologic Oncology Group study. Cancer. (1993) 71:1702–9. doi: 10.1002/cncr.2820710440 8381710

[3] Gadducci A Multinu F De Vitis LA Cosio S Carinelli S Aletti GD. Endometrial stromal tumors of the uterus: Epidemiology, pathological and biological features, treatment options and clinical outcomes. Gynecol Oncol. (2023) 171:95–105. doi: 10.1016/j.ygyno.2023.02.009 36842409

[4] Kommoss FKF Mar L-M Howitt BE Hanley K Turashvilli G Buslei R. High-grade endometrial stromal sarcomas with YWHAE::NUTM2 gene fusion exhibit recurrent CDKN2A alterations and absence of p16 staining is a poor prognostic marker. Mod Pathol. (2023) 36:100044. doi: 10.1016/j.modpat.2022.100044 36788095

[5] Seagle B-LL Shilpi A Buchanan S Goodman C Shahabi S. Low-grade and high-grade endometrial stromal sarcoma: A National Cancer Database study. Gynecol Oncol. (2017) 146:254–62. doi: 10.1016/j.ygyno.2017.05.036 28596015

[6] Pautier P Nam EJ Provencher DM Hamilton AL Mangili G Siddiqui NA. Gynecologic Cancer InterGroup (GCIG) consensus review for high-grade undifferentiated sarcomas of the uterus. Int J Gynecol Cancer. (2014) 24:S73–77. doi: 10.1097/IGC.0000000000000281 25341584

[7] Wu J Zhang H Li L Hu M Chen L Wu S. Prognostic nomogram for predicting survival in patients with high grade endometrial stromal sarcoma: a Surveillance Epidemiology, and End Results database analysis. Int J Gynecol Cancer. (2020) 30:1520–7. doi: 10.1136/ijgc-2020-001409 32839227

[8] Cabrera S Bebia V Acosta U Franco-Camps S Mañalich L García-Jiménez A. Survival outcomes and prognostic factors of endometrial stromal sarcoma and undifferentiated uterine sarcoma. Clin Transl Oncol. (2021) 23:1210–9. doi: 10.1007/s12094-020-02512-6 33210235

[9] Xue M Chen G Dai J Hu J. Development and validation of a prognostic nomogram for extremity soft tissue leiomyosarcoma. Front Oncol. (2019) 9:346. doi: 10.3389/fonc.2019.00346 31119101 PMC6504783

[10] Balachandran VP Gonen M Smith JJ DeMatteo RP. Nomograms in oncology: more than meets the eye. Lancet Oncol. (2015) 16:e173–180. doi: 10.1016/S1470-2045(14)71116-7 PMC4465353 25846097

[11] Amant F Floquet A Friedlander M Kristensen G Mahner S Nam EJ. Gynecologic Cancer InterGroup (GCIG) consensus review for endometrial stromal sarcoma. Int J Gynecol Cancer. (2014) 24:S67–72. doi: 10.1097/IGC.0000000000000205 25033257

[12] Cassier PA Lefranc A Amela EY Chevreau C Bui BN Lecesne A. A phase II trial of panobinostat in patients with advanced pretreated soft tissue sarcoma. A study from the French Sarcoma Group. Br J Cancer. (2013) 109:909–14. doi: 10.1038/bjc.2013.442 PMC3749588 23922114

[13] Yoon A Park J-Y Park J-Y Lee Y-Y Kim T-J Choi CH. Prognostic factors and outcomes in endometrial stromal sarcoma with the 2009 FIGO staging system: a multicenter review of 114 cases. Gynecol Oncol. (2014) 132:70–5. doi: 10.1016/j.ygyno.2013.10.029 24184602

[14] Liu H Zhang H Zhang C Liao Z Li T Yang T. Pan-soft tissue sarcoma analysis of the incidence, survival, and metastasis: A population-based study focusing on distant metastasis and lymph node metastasis. Front Oncol. (2022) 12:890040. doi: 10.3389/fonc.2022.890040 35875111 PMC9303001

[15] Kikuchi A Yoshida H Tsuda H Nishio S Suzuki S Takehara K. Clinical characteristics and prognostic factors of endometrial stromal sarcoma and undifferentiated uterine sarcoma confirmed by central pathologic review: A multi-institutional retrospective study from the Japanese Clinical Oncology Group. Gynecol Oncol. (2023) 176:82–9. doi: 10.1016/j.ygyno.2023.07.002 37478616

[16] Khan SR Soomar SM Asghari T Ahmed A Moosajee MS. Prognostic factors, oncological treatment and outcomes of uterine sarcoma: 10 years’ clinical experience from a tertiary care center in Pakistan. BMC Cancer. (2023) 23:510. doi: 10.1186/s12885-023-11000-3 37277708 PMC10243025

[17] Blay J-Y van Glabbeke M Verweij J van Oosterom AT Le Cesne A Oosterhuis JW. Advanced soft-tissue sarcoma: a disease that is potentially curable for a subset of patients treated with chemotherapy. Eur J Cancer. (2003) 39:64–9. doi: 10.1016/s0959-8049(02)00480-x 12504660

[18] Abu-Rustum N Yashar C Arend R Barber E Bradley K Brooks R. Uterine neoplasms, version 1.2023, NCCN clinical practice guidelines in oncology. J Natl Compr Canc Netw. (2023) 21:181–209. doi: 10.6004/jnccn.2023.0006 36791750

[19] Lewis D Liang A Mason T Ferriss JS. Current treatment options: uterine sarcoma. Curr Treat Options Oncol. (2024) 25:829–53. doi: 10.1007/s11864-024-01214-3 38819624

[20] Li D Yin N Du G Wang S Xiao Z Chen J. A real-world study on diagnosis and treatment of uterine sarcoma in Western China. Int J Biol Sci. (2020) 16:388–95. doi: 10.7150/ijbs.39773 PMC6990907 32015676

[21] Desar IME Ottevanger PB Benson C van der Graaf WTA. Systemic treatment in adult uterine sarcomas. Crit Rev Oncol Hematol. (2018) 122:10–20. doi: 10.1016/j.critrevonc.2017.12.009 29458779

[22] Kang N Zhang Y Guo S Chen R Kong F Wang S. Genomic and transcriptomic characterization revealed the high sensitivity of targeted therapy and immunotherapy in a subset of endometrial stromal sarcoma. Cancer Res Treat. (2023) 55:978–91. doi: 10.4143/crt.2022.1647 PMC10372608 36731460

[23] Somaiah N Conley AP Parra ER Lin H Amini B Solis Soto L. Durvalumab plus tremelimumab in advanced or metastatic soft tissue and bone sarcomas: a single-centre phase 2 trial. Lancet Oncol. (2022) 23:1156–66. doi: 10.1016/S1470-2045(22)00392-8 35934010

[24] Rauh-Hain JA del Carmen MG. Endometrial stromal sarcoma: a systematic review. Obstet Gynecol. (2013) 122:676–83. doi: 10.1097/AOG.0b013e3182a189ac 23921879

[25] van der Graaf WTA Blay J-Y Chawla SP Kim D-W Bui-Nguyen B Casali PG. Pazopanib for metastatic soft-tissue sarcoma (PALETTE): a randomised, double-blind, placebo-controlled phase 3 trial. Lancet. (2012) 379:1879–86. doi: 10.1016/S0140-6736(12)60651-5 22595799

