# Supplementary material for: Risk factors, survival analysis, and nomograms for high-grade endometrial stromal sarcoma patients with distant metastasis: a population-based study (2010–2019)
Source: Front Oncol. 2025 Mar 7;15:1567195. doi: 10.3389/fonc.2025.1567195 (PMC11925770; doi:10.3389/fonc.2025.1567195)

Figure. S1. Kaplan-Meier plots depicting overall survival in high-grade endometrial stromal sarcoma with distant metastasis (metastasis cohort) of N stage (A), surgery (B), chemotherapy (C), and systemic therapy (D).


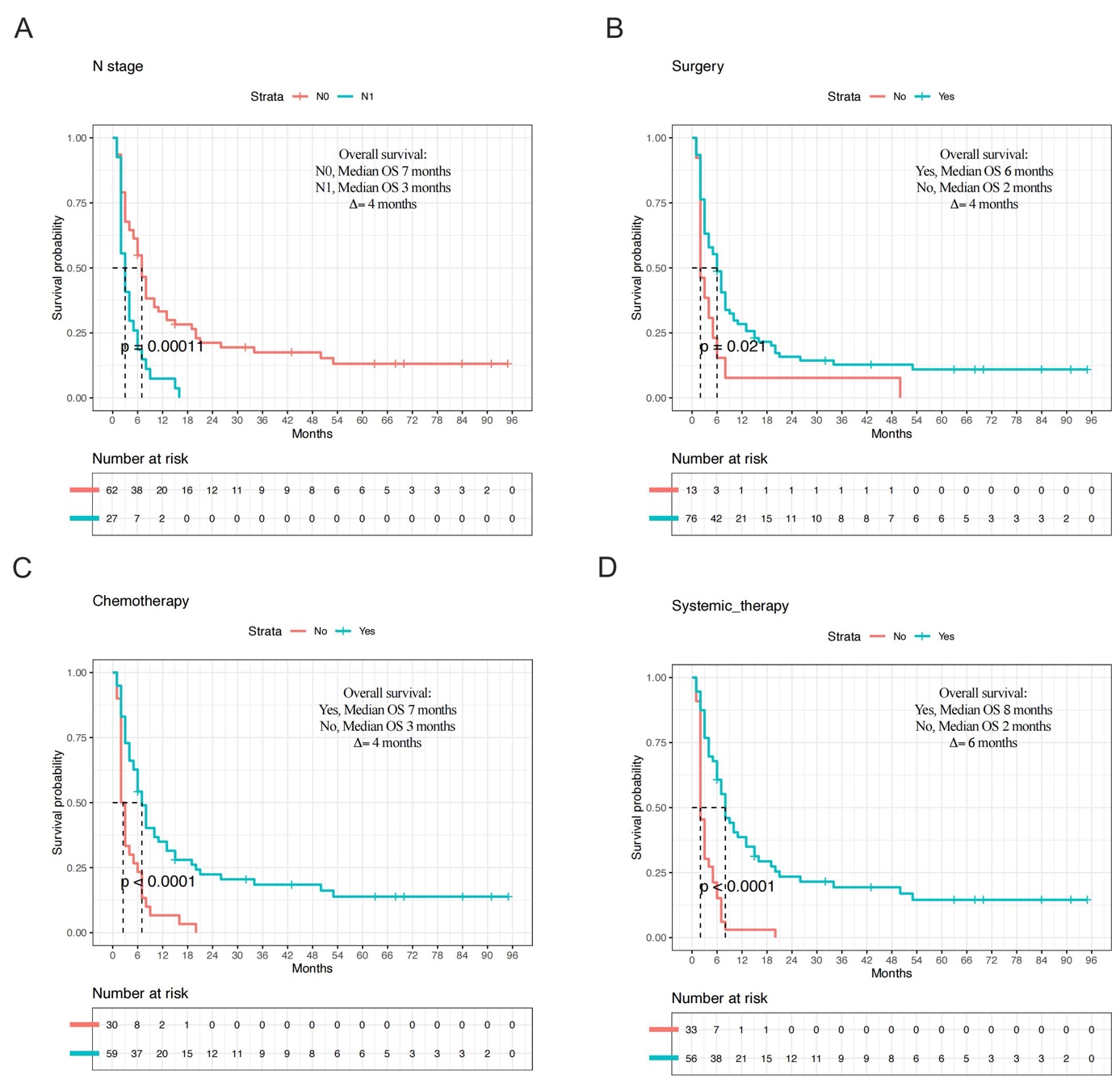

Supplement: Supplementary file 1 [file DataSheet1.docx]
